# Physiological Effects of Training in Elite German Winter Sport Athletes: Sport Specific Remodeling Determined Using Echocardiographic Data and CPET Performance Parameters

**DOI:** 10.3390/jcdd9080235

**Published:** 2022-07-25

**Authors:** Paul Zimmermann, Isabelle Schöffl, Volker Schöffl, Lukas Zimmermann, Max L. Eckstein, Othmar Moser, Jan Wüstenfeld

**Affiliations:** 1 Department of Cardiology, Klinikum Bamberg, 96049 Bamberg, Germany; paul.zimmermann@arcormail.de; 2 Interdisciplinary Center of Sportsmedicine Bamberg, Klinikum Bamberg, 96049 Bamberg, Germany; isabelle.schoeffl@me.com (I.S.); volker.schoeffl@me.com (V.S.); luki.z@web.de (L.Z.); 3 Division of Exercise Physiology and Metabolism, Department of Sport Science, University of Bayreuth, 95447 Bayreuth, Germany; max.eckstein@uni-bayreuth.de (M.L.E.); othmar.moser@uni-bayreuth.de (O.M.); 4 Department of Pediatric Cardiology, University Hospital Erlangen-Nuremberg, 91054 Erlangen, Germany; 5 Department of Traumatology and Orthopaedics, Klinikum Bamberg, 96049 Bamberg, Germany; 6 Department of Trauma Surgery, Friedrich Alexander Universität Erlangen-Nürnberg, 91054 Erlangen, Germany; 7 Section of Wilderness Medicine, Department of Emergency Medicine, University of Colorado School of Medicine, Aurora, CO 80045, USA; 8 School of Applied and Medical Sciences, Leeds Beckett University, Leeds LS1 3HE, UK; 9 Interdisciplinary Metabolic Medicine Research Group, Division of Endocrinology and Diabetology, Medical University of Graz, 8036 Graz, Austria; 10 Department Sports Medicine, Charité–Universitätsmedizin Berlin, Corporate Member of Freie Universität Berlin and Humboldt Universität zu Berlin, 14195 Berlin, Germany; 11 Insitute for Applied Training Science, 04109 Leipzig, Germany

**Keywords:** physiological demands, winter sport athlete, ski-mountaineering, biathletes, Nordic-cross country, echocardiography, cardiopulmonary exercise testing

## Abstract

Nine ski mountaineering (Ski-Mo), ten Nordic-cross country (NCC), and twelve world elite biathlon (Bia) athletes were evaluated for cardiopulmonary exercise test (CPET) performance and pronounced echocardiographic physiological cardiac remodeling as a primary aim of our descriptive preliminary report. In this context, a multicenter retrospective analysis of two-dimensional echocardiographic data including speckle tracking of the left ventricle (LV-GLS) and CPET performance analysis was performed in 31 elite world winter sports athletes, which were obtained during the annual sports medicine examination between 2020 and 2021. The matched data of the elite winter sports athletes (14 women, 17 male athletes, age: 18–32 years) were compared for different CPET and echocardiographic parameters, anthropometric data, and sport-specific training schedules. Significant differences could be revealed for left atrial (LA) remodeling by LA volume index (LAVI, *p* = 0.0052), LV-GLS (*p* = 0.0003), and LV mass index (LV Mass index, *p* = 0.0078) between the participating disciplines. All participating athletes showed excellent performance data in the CPET analyses, whereby significant differences were revealed for highest maximum respiratory minute volume (VE _maximum_) and the maximum oxygen pulse level across the participating athletes. This study on sport specific physiological demands in elite winter sport athletes provides new evidence that significant differences in CPET and cardiac remodeling of the left heart can be identified based on the individual athlete’s training schedule, frequency, and physique.

## 1. Introduction

The three described participating winter sport disciplines, ski-mountaineering (Ski-Mo), Nordic-cross country (NCC), and biathlon (Bia), are known to be very challenging for the individual athlete because they are performed in altitude environmental conditions and involve the whole body and uphill locomotion [1,2,3,4,5,6].

The individual sport performance of these athletes, especially the physiological demands and functional and structural cardiac remodeling have been reported in previous studies focusing on energy cost, training methods, training frequency, and pronounced structural and functional remodeling of the left heart by transthoracic echocardiographic assessment as well as cardiopulmonary exercise testing (CPET) performance data analyses [4,5,6,7,8,9,10,11,12].

Nevertheless, the outdoor sport community in general as well as the leisure winter sport community has been rapidly growing in the last decades and enjoys growing popularity. However, despite its popularity, sport-specific research about these professional athletes, particularly Ski-Mo, is up to now scarce [7,11,12]. As a consequence of the increasing professionalization of Ski-Mo from an unorganized leisure sport to a professional sport discipline, it will be represented as a winter sport discipline for the first time in the Milan-Cortina Olympic winter games in 2026 as stated by the International Olympic Committee with two disciplines (speed and individual) [7,13].

In the last decades, the term “athlete’s heart” has been defined by its individual structural, physiological, and functional sport-specific as well as electrophysiological remodeling based on cardiac imaging, CPET performance analysis, and clinical cardiological evaluation [12,14,15,16,17,18,19,20]. In this context, the sport-specific athlete’s background and training impact have to be taken into consideration for varying cardio-physiological adaption [12,14,16,20,21].

In our descriptive preliminary report on the physiological demands based on CPET performance data analyses as well as echocardiographic data assessment in German elite winter sport professionals, we elucidate for the first time an individual athlete’s sport-specific physiological adaption to the performed winter sport discipline. We are able to present significant physiological performance differences between the three different participating cohorts of athletes and to interpret the results against the individual athlete’s physique, training schedule, and training frequency. The matched data of the three participating cohorts represent the ideal comparison group for sport-specific cardiac remodeling, as their training impact and frequency as well as their sport physiological demands are comparable [12,22]. Our presented study on performance data analyses in elite winter sport athletes might contribute to individualizing athletes’ training schedules and their competition performance in the future and might pave the road to future scientific effort to strengthen the scientific basis of evidence.

## 2. Materials and Methods

### 2.1. Study Design

This was a multicenter retrospective data analysis of sport specific cardiac remodeling assessment by two-dimensional echocardiographic analysis focusing on the left heart including speckle tracking analysis and CPET performance data in 31 elite winter sport athletes, which were obtained in 2020 and 2021 during the annual medical sports medicine check-up [12,22]. The matched data of the elite winter sports athletes (14 women, 17 male athletes, age: 18–32 years) were compared for different echocardiographic and CPET performance parameters, athlete’s physique data, and sport-specific training aspects.

### 2.2. Participating Athletes

Thirty-one young elite winter sport professionals, all active members of the German National Team, participating in world championships as well as World Cups, were examined during the season of 2020/2021. During the severe worldwide COVID-19 pandemic situation, no participating athlete was infected or had to be excluded due to post-COVID-19 infection syndromes.

All participants (14 women, 17 male athletes, age: 18–32 years) were assessed by an individual sports medicine check-up in their supervising sports medicine performance center—the Interdisciplinary Center of Sportsmedicine, Klinikum Bamberg or the Institute for Applied Exercise Science, Leipzig. All participating athletes showed comparable anthropometric data, whereby male athletes weighed 65–81 kg, had a body mass index (BMI) of 18–25 kg/m^2^, and had a height of 175–186 cm. The participating female athletes weighed 47–69 kg, showed a BMI of 18–23 kg/m^2^, and had a height of 154–176 cm, as reported before by Zimmermann et al. [12,22]. All participants were professional winter sport athletes with a total amount of 20–25 training hours per week during the season—based on the individual high volume training schedule—and performed up to 10 trainings hours in the season’s recreational time, including functional strength training (ST) units, continuous endurance training (ET) such as running and cycling, and individual training units to improve muscle disbalances, as pictured in Table 1. No participating athlete had to be excluded from our study due to adverse cardiac events or arrhythmias in any individual athlete’s history. Due to the anthropometric variability with Ski-Mo athletes representing the youngest and physically smallest athletes, and variable fewer lifetime training hours and training schedules, across the three participating winter sport athletes an age-matched analysis is not possible.

### 2.3. Echocardiographic Assessment and CPET Performance Analysis

During the annual sports medicine evaluation in the accompanying performance center, we performed twelve-lead electrocardiograms (ECGs), two-dimensional echocardiographic examination including left ventricle-global longitudinal strain (LV-GLS), and CPET performance analysis in all participating elite winter sport athletes.

The twelve-lead ECG was performed in a lying position with 50 mm/s (CardioSoft V6.73, GE Medical Systems, Munich, Germany and Custo med cardio, Custo med GmbH, Ottobrunn, Germany) to define resting heart rate in beats per minute (bpm) after 5 min. Anthropometric data were additionally evaluated for all athletes, such as body mass index (BMI in kg/m^2^), body surface area (BSA in m^2^), and resting blood pressure level (in mmHg) after resting for five minutes in a supine position.

As part of the sports medicine checkup, the echocardiographic functional and morphological assessment was performed using a commercially available echocardiographic system Phillips EPIQ 7 device with an X5-1 Matrix-array transducer (Phillips Healthcare, Eindhoven, The Netherlands), following a standard protocol as described before [12,23]. The acquired images were analyzed and stored digitally; for measurements, sequences of at least three heart beats were stored and analyzed [12]. Our participants were evaluated by two-dimensional echocardiographic analyses performed according to the general recommendations [12,23,24,25]. The systolic LV ejection fraction (LV-EF) was evaluated using biplane Simpson rule, based on the apical two- as well as the apical four-chamber view and using Teichholz method ejection fraction calculation in the parasternal long axis. For both atria and both ventricles, two-dimensional linear dimensions assessment was performed according to the recommendations [23,24,25]. A morphological and functional analysis of the right heart, including an estimation of the right ventricular (RV) systolic function using the TAPSE (tricuspid annular plane systolic excursion) was performed in the apical four-chamber view.

Furthermore, based on the two-dimensional echocardiographic measurements of the left heart, specific calculations for each participating athlete were performed for the following indexes by an individual specific validated method: the left ventricular mass index (LV Mass index) by a validated method [26], the relative wall thickness (RWT) of the left ventricle (LV) based on the formula (2× posterior wall thickness)/left ventricle enddiastolic diameter (LVedd) [26], and the left atrial volume index (LAVI) by a validated method [25].

For functional assessment of the LV, the LV diastolic function was assessed by the pulse-wave Doppler in the apical four-chamber view referring to the peak early filling (E wave) and late diastolic filling (A wave) velocities. To quantify the peak early velocity E’, a tissue Doppler imaging of the lateral mitral anulus in the apical four chamber view was performed [12,23,25].

To reveal the individual athlete’s LV-GLS pattern by two-dimensional strain in the apical views, we performed LV speckle tracking analysis and focused on the LV and did not evaluate the RV and LA strain pattern. Therefore, the Philips QLAB cardiac analysis application “AutoStrain” was used (Phillips Healthcare, Eindhoven, The Netherlands). Each athlete was evaluated for the prevalence of left and right heart valve regurgitation during each individual athlete’s standard echocardiographic assessment.

The CPET performance analyses were conducted in accordance with the recommendations of the American Heart Association (AHA) [27], and as predetermined by the national winter sport discipline association, either on a bicycle or on a treadmill. Therefore, the CPET step-wise protocol started with a workload of 80 Watts increasing the workload per 40 Watts every 3 min or alternatively starting with 100 Watts increasing per 30 Watts every 3 min until volitional exhaustion. Alternatively, the treadmill tests started with a workload of 10 km/h for male and 8 km/h for female athletes for 3 min and then increasing the speed by 1.0 km/h or 1.5 km/h every 3 min, as described in our previous reporting on CPET [22]. All performance data from the CPET, twelve-lead ECG, and blood pressure were recorded continuously. To define individual athletes’ peak performance criteria for CPET analysis, attention was paid to several performance parameters. Primarily, each participant was observed to define individual peri- and post-exercise lactate level with a capillary blood analysis from the earlobe at each workload step, individual anaerobic threshold (4 mmol/L), and their recovery time (maximum 15 mmol/L) [22]. Secondly, several additional peak performance parameters were recognized: a respiratory exchange rate (RER) of 1.15 at peak performance, reaching 85% of the individual maximum predicted heart rate (220 bpm minus age in years), leveling off of the VO_2 maximum_, and individual assumed exercise time of CPET duration. Each athlete was evaluated for the specific athlete’s exertion level by the Borg RPE scale (Values ≥ 17). In summary, to define an individual athlete’s maximal CPET effort, a minimum of three of the above-mentioned criteria were taken into consideration [22]. Next to the recorded CPET performance data, the oxygen pulse at VT2 (Oxygen pulse VT2) as well as the peak oxygen pulse (Oxygen pulse _maximum_) were assessed by dividing the derived VO_2_ by the heart rate at VT2 or the maximum heart rate [28].

To summarize the obtained data from the echocardiographic and CPET evaluation, additional information for each participating athlete about the training schedules and frequency was collected from the responsible national winter sport discipline association. In particular, detailed information about the individual athletes’ training component and frequency was provided to understand the distinctions between our three participating cohorts of world elite winter sports athletes.

### 2.4. Statistical Analysis

Our data were analyzed with Graph Pad Prism 8.2.1(279) (Graph Pad Software; San Diego, CA, USA). Firstly, all acquired data were assessed for normal distribution by analyzing the data by means of Kolmogorov–Smirnov normality testing. Afterwards, using non-parametric Kruskal–Wallis, we tested our numerical data group comparisons for nine Ski-Mo athletes (5 male, 4 women), ten NCC athletes (6 male, 4 women), and twelve elite Bia athletes (6 male, 6 women). Afterwards, a gender-specific analysis for the interesting echocardiographic and CPET parameters was utilized equally by using non-parametric Kruskal–Wallis testing. *p* ≤ 0.05 was accepted as statistically significant. Since the manuscript is focusing on a clinical scientific outcome in a small limited number of world elite winter sport professionals, the sample size is not calculated in our preliminary reporting.

### 2.5. Ethical Consideration

The study protocol (17_21 B) was approved by the local ethics committee of the University of Nurnberg-Erlangen. In general, the study was conducted in conformity with the declaration of Helsinki and Good Clinical Practice [29]. Prior to any trial-related activities and measurements, our participating athletes gave their written informed consent and were informed about the study protocol and the following measurements.

## 3. Results

### 3.1. Baseline Characteristics and Anthropometric Data

In our descriptive preliminary report, a total of 31 young professional winter sports athletes, including Ski-Mo, NCC, and Bia athletes, were examined. The matched data of the three different participating cohorts were compared for different anthropometric data, for morphological and functional sport-specific cardiac remodeling by echocardiographic assessment and CPET performance parameters. For the echocardiographic assessment, we examined one more male Ski-Mo athlete, who felt too unwell to perform CPET and was therefore solely analyzed for sport specific echocardiographic remodeling, as presented below. The recorded athletes’ heart rate at baseline (bpm), individual heart rate response at VT2 as well as at maximum effort during CPET, the resting blood pressure level (mmHg), height, weight, BMI, and BSA are represented in Table 2, adapted to Zimmermann et al. [12,22].

### 3.2. Morphological and Functional Cardiac Remodeling

Evaluating the morphological and functional cardiac remodeling of our participating elite winter sport athletes, we could reveal significant differences between the three different winter sport disciplines (results shown in Table 3).

In the two-dimensional echocardiographic examination, all participating elite winter sport athletes showed a normal to little reduced systolic LV ejection fraction (LV-EF) estimated by the biplane Simpson and Teichholz rule. For all three different winter sport professionals, we calculated the LV Mass Index as an indexed parameter and obtained significantly higher values for this parameter for NCC and Bia athletes compared to Ski-Mo athletes (results shown in Table 3). Additionally, significant higher relative wall thickness (RWT) could be shown in NCC and Bia athletes in comparison to Ski-Mo professionals (results shown in Table 3).

**Figure 1:**
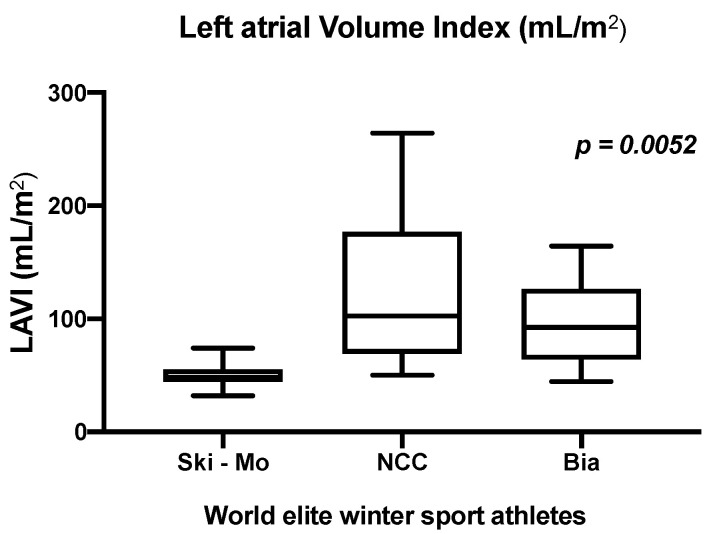
Analysis of the left atrial volume index (LAVI)—significant different results defined by the athletic sporting discipline in world elite winter sport professionals (*p* = 0.0052), modified from Zimmermann et al. 2021 [12].

Analyzing the morphological structure of the left atrial remodeling, significant differences could be revealed across our three participating athletes. The LAVI (mL/m^2^), as an indexed parameter, was significantly enlarged in NCC and Bia athletes compared to Ski-Mo professionals (Figure 1). This was also true for the sex-related sub-analyses. In detail, male and female Ski-Mo athletes showed the smallest values (results shown in Table 3).

Focusing the left ventricular remodeling, especially the eccentric remodeling of the LV, such as interventricular septal wall diameter (IVSd), the left ventricular posterior wall diameter (LVPWd), and the RWT, we could elucidate significant differences across our participating athletes. The NCC and Bia athletes in general as well as in the gender-specific analyses showed significantly thicker LV wall diameter than Ski-Mo athletes. No significant structural anatomic differences between our athletes could be proven for LVedd, for the end-diastolic volume (LV EDVedd), the right heart dimensions as right atrial end-systolic diameter (RA endsyst), and right ventricular end-diastolic size (RV edd) as well as the TAPSE of RV.

**Figure 2:**
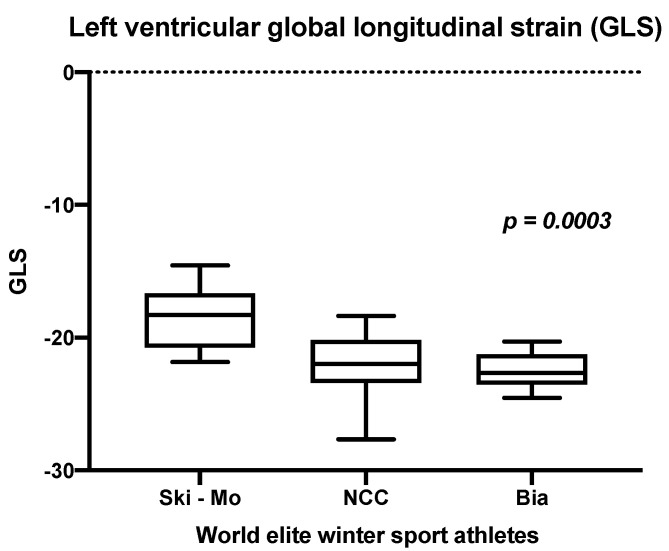
Analysis of the left ventricular global longitudinal strain (GLS) in world elite winter sport professionals (*p* = 0.0052), modified from Zimmermann et al., 2021 [12].

Focusing on the functional cardiac remodeling, i.e., diastolic function and speckle tracking analysis in our elite winter sport athletes, we were able to prove significant differences for the E/A ratio as criteria for LV diastolic function, but no significant differences in the gender sub-analysis (results shown in Table 3). The speckle tracking analysis with the main emphasis on the LV-GLS in our athletes revealed significant differences with regards to the background of the sport specific discipline in our small cohort. The LV-GLS in male athletes with Ski-Mo athletes had the lowest values (*p* = 0.0003, Figure 2 and results shown in Table 3).

### 3.3. Sport-Specific Physiological Performance by Laboratory CPET Analyses

Next to the presented anthropometric data and echocardiographic assessment, our professional winter sport athletes were compared for CPET performance parameters, whereby all participating athletes showed excellent performance data as presented in Table 4 (adapted from Zimmermann et al., 2022 [22]). Updated Olympic-medal-level performance benchmark data were used as reference [30].

Analyzing the sport specific aerobic capacity in our participating athletes, the highest maximum respiratory minute volume (VE _maximum_) was elucidated for Bia athletes, who showed significantly higher values in comparison to Ski-Mo athletes (results presented in Table 4).

No significant differences could be revealed for the maximum ventilatory oxygen uptake (VO_2 maximum_) nor for the indexed ventilatory oxygen uptake (VO_2_) at the maximum performance level (VO_2_/kg _maximum_) across the three participating winter sport professionals.

**Figure 3:**
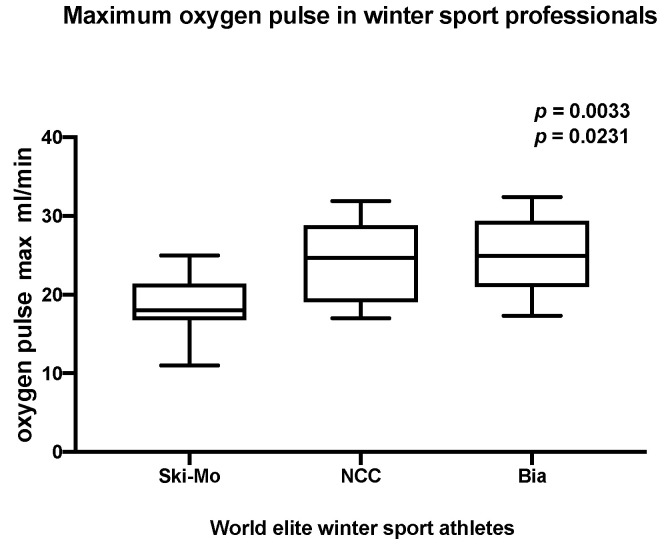
Analysis of maximum oxygen pulse in world elite winter sport professionals (*p* = 0.0033, *p* = 0.0231), modified from Zimmermann et al., 2022 [22].

Analyzing the Oxygen pulse _maximum_ as presented in Table 4, NCC and Bia athletes showed significantly higher performance values than our participating Ski-Mo athletes (Figure 3).

Focusing on the gender-specific CPET performance data analyses, the female Bia athletes were able to assume the highest peak oxygen pulse (Oxygen pulse _maximum_) performance parameters across the three winter sport groups (*p* = 0.0190).

**Figure 4:**
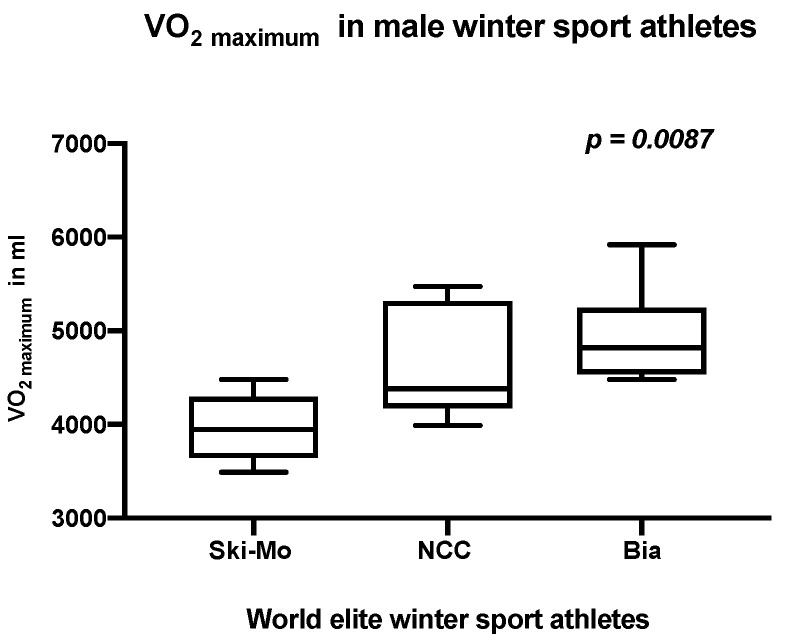
Analysis of maximum ventilatory oxygen uptake (VO_2 maximum_) in male world elite winter sport professionals (*p* = 0.0087).

Additionally, in our male winter sport professionals, the highest maximum respiratory minute volume (VE _maximum_, *p* = 0.0087), the highest maximum ventilatory oxygen uptake (VO_2 maximum_, *p* = 0.0087, Figure 4), and the best peak oxygen pulse (Oxygen pulse _maximum_, *p* = 0.0260) were highlighted for the male Bia athletes in comparison to the other two participating sport disciplines (results shown in Table 4).

## 4. Discussion

In the present descriptive preliminary report, morphological and functional cardiac remodeling as well as sport specific CPET performance parameters in German world elite winter sport athletes were investigated and compared with each other for the first time. The investigated winter sports in this study are known for their high energy demands, involve the whole body, and are often performed at altitude [3,4,5,9,10]. Environmental conditions as well as exceptionally high aerobic turnover, an excellent anaerobic power, and different race speed qualities have to be taken into consideration as variable parameters influencing individual athletes’ performance [8,15,22,31,32,33,34].

In this context, repeated intensity fluctuations and physiological adaptions have been reported before in cross-country skiing [33]. These results emphasize the impact of the high endurance demand in our analyzed winter sports on sport-specific echocardiographic remodeling as well as physiological response assumed by CPET parameters.

The impact of two-dimensional echocardiography on morphological and functional cardiac remodeling of the athlete’s heart has been studied and described before. Morphological features, especially left heart remodeling with enlarged heart structures in male athletes in general are the main findings [35].

Classifying our observed descriptive findings in this context, several influencing factors have to be taken into consideration. The training schedule and frequency show inter- group differences. These differences, especially the individual training strategies, focusing on ET or ST components, might slightly and rather likely in long-term perspective contribute to individualized variable sports specific adaptions. Analyzing the baseline morphological and functional echocardiographic remodeling across our three participating elite winter sport disciplines, we could reveal significant differences in the NCC and Bia sports in comparison to Ski-Mo athletes. The Ski-Mo athletes, representing the youngest and physically smallest athletes, with less lifetime training hours and the highest amount of endurance training (ET) during training schedule [4,12], revealed significantly lower values for LV mass index for Ski-Mo compared to NCC and Bia athletes. In athletes focusing on ST, higher LV wall thickness can be observed [12,36]. In our study, we elucidated LA remodeling especially in NCC and Bia athletes by the analyzed indexed parameter—LAVI. Significantly higher values were observed for the NCC and Bia athletes compared to Ski-Mo athletes with less life time training hours. Nevertheless, in elite endurance athletes, LA remodeling has been reported as a typical characteristic and might contribute to a transient balanced cardiomyopathy with the further risk of developing an atrial cardiomyopathy [37]. In the end, these descriptive echocardiographic findings do not translate to an increased VO_2_/kg _maximum_ as the reference parameter for peak performance in our NCC and Bia athletes. Nevertheless, we could elucidate significantly higher oxygen pulse _maximum_ levels in Bia and NCC athletes, which might be suggestive for enhanced peak performance. This enhanced peak performance—displayed by higher oxygen pulse _maximum_ levels—might be assumed by varying morphological and functional cardiac remodeling across our three groups. Regarding this parameter, it has to be stated clearly that several parameters and circumstances might influence this parameter next to the analyzed baseline echocardiographic parameters at rest. Interindividual exercise-dependent blood pressure increase, variable hemoglobin levels, mild right–left atrial shunt, mild intrapulmonary shunting with oxygenation mismatch, or variable dynamic stroke volume determined by alternating volume preload conditions, transient balanced sport specific atrial remodeling, or variable autonomous vagal regulation in athletes are previously known influencing factors [38,39]. These mentioned influencing factors have to be taken additionally into consideration judging the athlete’s peak performance. Our athletes’ performance assessment based on resting echocardiographic assessment and CPET performance analysis has to be interpreted with caution and displays only partly relevant influencing parameters for athletes’ race performance. Transient balanced sport-specific atrial remodeling might contribute to an improved exercise capacity and cardiac output during exercise in athletes revealed by positive correlations between VO_2 maximum_ and LA passive emptying fraction [40] as well as mild association between peak cardiac performance output and resting left heart cardiac parameters [41].

Analyzing the controversially discussed topic of functional LV remodeling by E/A and E/E′ ratio observation, our data in world elite winter sport professionals revealed comparable results in elite athletes with previous research [16,35]. Various factors influencing diastolic function have been reported before, such as low resting heart rate, increased vagal tone, and improved hemodynamic filling of the LV in athletes [16]. Highlighting the impact of LV-GLS analysis in winter sport professionals, we revealed slightly reduced values in Ski-Mo compared to NCC and Bia athletes. By using LV-GLS observation, the distinction between inherited or acquired cardiomyopathies and pronounced physiological cardiac remodeling can be improved. Whereas the normal LV-GLS strain range is estimated to be between −18% and, −25%, strain analysis in general can detect functional abnormalities and early changes in cardiac mechanics long before structural damages can be revealed [12,42,43,44].

Our findings have to be interpreted carefully and are limited due to several influencing circumstances. On the one hand, the impact of various athletes’ anthropometric data, different training schedule, and frequency, and on the other hand the data acquirement in the preseason preparation time as well as an interobserver variability might have contributed to the observed differences [12]. Nevertheless, our descriptive results might emphasize the impact of pronounced specific training-induced cardiac remodeling in athletes. While the clinical atrial cardiomyopathy is difficult to objectify, an increased risk for future degeneration to a pathological entity within the lifetime of a sports career might be assumed [12].

We evaluated the sport-specific physiological performance of professional winter sport athletes with laboratory CPET analyses as predictors of performance. Thus, we were able to elucidate different “adaption patterns” related to the impact of sport discipline and training schedule. First of all, Ski-Mo athletes are known for their enhanced aerobic capacity due to repeated intensity fluctuations, high intensity sprints, uphill locomotion, and high aerobic energy turnover [4,7,13,22,45]. Our data indicate similar findings with a comparable VO_2 maximum_ but a lower VE _maximum_ and a lower cardiac output, determined by a lower Oxygen pulse _maximum_. Our findings are supported by previous data, which revealed a positive correlation between increasing age and maximum oxygen uptake and anaerobic threshold in German Nordic combined athletes [46]. An improved development of VO_2 maximum_ in relation to athlete’s age and training conditions, i.e., training volume and lifetime training hours, was also noted in NCC athletes [47].

Summarizing the obtained results from our descriptive reporting study, we were able to detect valuable parameters for significant sport specific cardiopulmonary adaption in participating winter sport athletes. Although it might be difficult to derive reliable conclusions in this small sample size of world elite winter sport professionals, the reported CPET performance data and sport specific cardiac remodeling in the physically stronger NCC and Bia athletes might contribute to an enhanced peak performance of these two established winter sport disciplines. On the one hand, the participating athletes did not differ significantly with regard to the indexed ventilatory oxygen uptake at the maximum performance level (VO_2_/kg _maximum_), but additionally analyzing the oxygen pulse _maximum_, NCC and Bia athletes showed significantly higher peak performance values than our participating Ski-Mo athletes. Carefully interpreting these obtained descriptive findings, we might assume the described physiological adaption as well as sport specific echocardiographic remodeling, displayed by higher LV Mass index, larger left atrial remodeling as measured by LAVI, and higher values for the LV-GLS in NCC and Bia athletes. These obtained data and their drawn logical conclusion remain, in the end, speculative, limited by the small sample size of world elite winter sport professionals. Regarding the sport-specific left heart remodeling in interaction with the above-described individual athlete’s physiological adaption, an enhanced cardiac and physiological performance in NCC and Bia athletes—especially at maximum effort—might be assumed. These obtained findings will have to be confirmed in larger study populations and long-term follow-up observation in cooperation with the supervising national team staff and might contribute to individual sport-specific training planning in these elite winter sport professionals in the future.

Our study has several limitations as mentioned above in the discussion. Firstly, the number of participating elite winter sport athletes is relatively small as we enrolled only high-level athletes from the German national teams competing at World Cups and world-class events. Secondly, the echocardiographic assessment and CPET performance data of the participating athletes were assessed in a multicenter study design, implying a certain interobserver variability with respect to data acquirement. Thirdly, our performance measurements were acquired in the preseason preparation time in summer in a multicenter design with respect to a deviation in individual training schedules resulting in inter-group heart volume and training intensity variability. Furthermore, the anthropometric variability due to Ski-Mo athletes representing the youngest and physically smallest athlete category with fewer lifetime training hours might contribute to an interindividual variability evaluating sport specific cardiac remodeling and physiological adaption in these athletes. Additionally, the mixture of young and experienced NCC and Bia athletes entails an intra-cohort variability and contributes to a certain standard deviation in our cardiac and CPET measurements. Last, we focused on speckle tracking of LV-GLS and not on the circumferential LV strain analysis. We performed no specific strain analysis in the RV and LA, which has to be stated as an important limitation of this study. Taking these circumstances into consideration, we agree that our paper should be likely regarded as an interesting descriptive preliminary reporting for sport-specific remodeling in elite German winter sport athletes. Future research might focus on larger athlete sample sizes to specify the presented descriptive findings with statistical adjustment for anthropometric baseline characteristic, athlete’s age, lifetime training hours adjustment, or detailed training schedule adjustment.

## 5. Conclusions

This descriptive reporting provides new evidence that in different German world elite winter sport professionals, significant differences in morphological and functional remodeling of the left heart as well as for CPET parameters can be demonstrated, against the background of athlete’s anthropometric data, athlete’s physique, and training components and frequency.

Our results have to be handled with care due to the mentioned limitations and might serve as a preliminary report. Therefore, our results analysis—in general as well as in the gender-specific subgroup analyses—can identify physiological differences in morphological and functional sport specific cardiac remodeling. This was revealed in the speckle tracking analysis, focusing the LV-GLS, LV mass index parameters, and LA remodeling as measured by LAVI. On the other hand, sport-specific individual differences in the CPET performance can be elucidated for our three participating cohorts, especially due to the maximum performance parameters, such as VE _maximum_ and Oxygen pulse _maximum_.

These obtained differences between the three participating groups might define a pronounced athlete’s individual structural and functional sport specific cardio-physiological adaption. Nevertheless, when interpreting an athlete’s heart by echocardiographic assessment and individual CPET performance data analysis, the impact of the athlete’s physique, training schedule, and frequency have to be taken into consideration. From this aspect, our descriptive reporting might pave the road to future studies with greater number of participating athletes and long-term follow-up to verify the impact on sport-specific athletes’ heart adaption and to further strengthen the scientific evidence base.

## Figures and Tables

**Table 1:** Baseline training schedule in winter sport professionals.

Athlete	Average Years of Training	Pre-Season			In-Season		
		10 Training Hours per Week			20–25 Training Hours per Week		
		Endurance	Strength	Movement Specific/Flexibility	Endurance	Strength	Movement Specific/Flexibility
Ski-Mo	5 ± 3	90%	5%	5%	90%	7%	3%
NCC	15 ± 5.3	84%	10%	6%	89%	8%	3%
Bia	14 ± 4.5	76%	11%	13%	87%	9%	4%

Abbreviations: Ski-Mo, Ski-mountaineering; NCC, Nordic Cross-Country; Bia, Biathletes.

**Table 2:** Baseline winter sport professional characteristics.

	Ski-Mo *n* = 9		NCC *n* = 10		Biathletes *n* = 12	
	Male	Female	Male	Female	Male	Female
	*n* = 5	*n* = 4	*n* = 6	*n* = 4	*n* = 6	*n* = 6
Age (y)	21.4 ± 1.8	20.8 ± 2.4	26.3 ± 4.1	25.5 ± 0.5	27.3 ± 3.6	29.0 ± 3.2
Height (cm)	178.0 ± 3.9	163.5 ± 8.8	181.3 ± 4.7	171.2 ± 5.8	180.9 ± 5.1	172.8 ± 3.7
Weight (kg)	66.5 ± 0.8	53.2 ± 6.5	72.0 ± 3.0	63.4 ± 5.9	77.1 ± 3.7	62.5 ± 4.1
Resting blood pressure	118 ± 5.4	100 ± 8.2	125 ± 8.3	105 ± 7.2	117 ± 7.6	108 ± 6.2
systolic/diastolic (mmHg)	78 ± 4.0	72 ± 1.5	78 ± 2.9	71 ± 3.8	77 ± 2.2	70 ± 3.3
Resting heart rate (bpm)	41 ± 4.6	44 ± 4.5	42 ± 3.6	46 ± 5.1	41 ± 4.2	45 ± 5.1
Heart rate VT2 (bpm)	133 ± 22	132.3 ± 1.9	136.3 ± 11.6	128.3 ± 9.1	148.5 ± 20.9	134.5 ± 8.1
Maximum heart rate (bpm)	185.6 ± 6.3	171.8 ± 2.5	183 ± 14.3	173.8 ± 4.0	179.5 ± 10.3	181.0 ± 12.9
BMI (body mass index in kg/m^2^)	19.9 ± 1.4	19.8 ± 0.4	22.0 ± 1.1	22.0 ± 1.1	23.6 ± 0.9	20.9 ± 1.0
BSA (body surface area in m^2^)	1.70 ± 0.06	1.61 ± 0.12	1.88 ± 0.04	1.81 ± 0.07	1.92 ± 0.04	1.77 ± 0.05

Data are presented as mean with standard deviation Abbreviations: y, years; cm, centimeter; kg, kilogram; bpm, beats per minute; m^2^, square meter

**Table 3:** Echocardiographic Measurements in elite winter sport athletes, adapted from Zimmermann et al., 2021 [12].

	Ski-Mo (I) *n* = 10		NCC (II) *n* = 10		Biathletes (III) *n* = 12		*p*-Value
	Male Female		Male Female		Male Female		
	*n* = 6	*n* = 4	*n* = 6	*n* = 4	*n* = 6	*n* = 6	
**LV edd (mm)**	50.83 ± 4.22	45.25 ± 5.96	55.50 ± 3.83	50.75 ± 3.50	55.50 ± 5.24	49.50 ± 1.52	ns
	**48.6 ± 5.48**		**53.6 ± 4.27**		**52.5 ± 4.83**		
**LV Mass Index (g/m)**	97.2 ± 25.2	76.3 ± 26.7	130.7 ± 16.5	106 ± 16.4	133.5 ± 20.6	102.3 ± 14.8	0.0078 *
	−88.8 ± 26.6 *		120.8 ± 20.1 *		117.9 ± 23.6 *		
**Relative wall Thickness RWT**	0.38 ± 0.03	0.34 ± 0.06	0.40 ± 0.04	0.41 ± 0.04	0.40 ± 0.04	0.42 ± 0.04	Ski-Mo vs. NCC 0.0230 * Ski-Mo vs. Bia 0.0230 *
	**0.37 ± 0.05**		**0.41 ± 0.03**		**0.41 ± 0.04**		
**IVSd (mm)**	8.67 ± 1.97	8.25 ± 2.50	11.00 ± 0.63	10.50 ± 0.58	10.83 ± 0.98	9.67 ± 1.37	Ski-Mo vs. NC C 0.0266 * Ski-Mo vs. Bia 0.0337 *
	**8.5 ± 2.07**		**10.4 ± 1.17**		**10.3 ± 1.29**		
**LVPWs (mm)**	3.97 ± 11.03	7.75 ± 1.50	11.17 ± 0.41	10.50 ± 0.58	12.33 ± 2.07	10.17 ± 1.17	Ski-Mo vs. NC C 0.0161 * Ski-Mo vs. Bia 0.0030 *
	**8.9 ± 1.52**		**10.9 ± 0.57**		**11.3 ± 1.96**		
**E/A**	2.18 ± 0.58	1.98 ± 0.17	2.48 ± 0.26	2.40 ± 0.77	1.97 ± 0.52	1.75 ± 0.40	NCC vs. Bia 0.0166 *
	**2.1 ± 0.45**		**2.5 ± 0.49**		**19 ± 0.46**		
**E/E’**	6.75 ± 1.71	7 ± 1.79	6.80 ± 0.86	6.13 ± 1.22	7 ± 0.86	6.37 ± 1.04	ns
	**6.9 ± 1.66**		**6.4 ± 1.09**		**6.7 ± 0.97**		
**LAVI (mL/m^2^)**	51.83 ± 12.1	46.25 ± 11.1	150 ± 84.58	89.3 ± 45.7	117.5 ± 37.7	72.8 ± 19.6	0.0052 *
	**49.6 ± 11.4**		**125.7 ± 75.2**		**95.2 ± 36.9**		
**RA (cm^2^)**	19.17 ± 3.87	16.75 ± 2.87	24.83 ± 3.73	18.28 ± 4.72	20.78 ± 3.64	15.50 ± 2.40	ns
	**18.2 ± 3.55**		**22.2 ± 5.16**		**18.1 ± 4.03**		
**GLS**	−18.26 ± 2.21	−18.83 ± 2.93	21.21 ± 1.99	−23.25 ± 3.23	22.62 ± 1.26	22.34 ± 1.42	0.0003 *
	**−18.5 ± 2.38**		**−22.0 ± 2.61**		**−22.5 ± 1.29**		

Data are presented as mean with standard deviation. *p* value *, statisticallly significant (*p* < 0.05). Abbreviations: LV edd, left ventricle enddiastolic size; LV, left ventricular; IVSd, interventricular septal wall thickness at diastole; LVPWd, left ventricular posterior wall thickness at diastole; E/A and E/E, parameters for diastolic function of the left ventrile; LAVI, left atrial volume index; RA, right atrum; GLS, global longitudinal strain; ns, non-significant.

**Table 4:** Cardiopulmonary Exercise Testing (CPET) performance parameters in elite Winter Sport professionals, adapted from Zimmermann et al., 2022 [22].

	Ski-Mo (I)		NCC (II)		Biathletes (II)		*p*-Value Male	*p*-Value Female	Overall *p*-Value
	Male	Female	Male	Female	Male	Female			
VE _maximum_ (L)	134.9 ± 24.6	109.2 ± 20.6	166.2 ± 28.4	118 2 ± 23.8	175.8 ± 11.7	125.4 ± 9.1	Ski-Mo vs. Bia 0.0087 *	ns	Ski-Mo vs. Bia 0.0409 *
	**123.5 ± 25.4**		**147.0 ± 3 5.4**		**150.6 ± 28.1**				
VO_2 maximum_ (mL)	3964.8 ± 1367.8	3021.3 ± 515.1	4620.8 ± 603.8	3315. 3 ± 576.0	4935.2 ± 525.1	3555. 7 ± 274.7	Ski-Mo vs. Bia 0.0087 *	Ski-Mo vs. Bia 0.0381 *	**ns**
	**3545.4 ± 643.7**		**4098.6 ± 876.2**		**4245. 4 ± 823.8**				
VO_2_/kg _maximum_ (mL/kg)	65.0 ± 7.9	57.4 ± 4.5	64.5 ± 7.1	52.7 ± 4.9	64.6 ± 4.4	57.4 ± 2.3	**ns**	**ns**	**ns**
	**61.6 ± 7.5**		**59.7 ± 8.6**		**61.0 ± 5.0**				
Oxygen pulse _maximum_ (mL/min)	20.8 ± 30	15.6 ± 31	26.9 ± 4.2	19.4 ± 31	27.8 ± 3.2	22. 8 ± 5.4	Ski-Mo vs. NCC 0.0303 * Ski-Mo vs. Bia 0.0260 *	Ski-Mo vs. Bia 0.0190 *	Ski-Mo vs. NCC 0.0231 * Ski-Mo vs. Bia 0.0033 *
	**18.5 ± 4.0**		**23.9 ± 5.3**		**25. 3 ± 4.9**				

Data are presented as mean with standard deviation. *p* value *, statistically significant (*p* < 0.05). VO/kg, ventilatory oxygen uptake per kilogram; L liter; mL, milli-liter; min, minute; ns, not significant. Abbreviations: CPET, cardiopulmonary exercise testing; Ski-Mo, Ski-mountaineering; NCC, Nordic Cross-Country; VE, respiratory minute volume; VO, ventilatory oxygen uptake.

## Data Availability

Individual anonymized data supporting the analyses of this study contained in this manuscript will be made available upon reasonable written request from researchers whose proposed use of data for a specific purpose has been approved.
